# Hæmatology—IX

**Published:** 1910-10-29

**Authors:** 


### PATHOLOGY.

### HÆMATOLOGY—IX.

### LEUKÆMIA, ETC.

Leukæmia, also known as leucocythæmia, is a condition where the blood contains an abnormal excess of white cells. It may be classified as follows:—

Leukæmia { Spleno-medullary or myelogenic { acute / chronic; Lymphatic { acute / chronic

I. *Acute spleno-medullary or myelogenic leukæmia.*—Billings and Cap, two Americans, brought this somewhat rare form of the disease into prominence some years ago. They described one case, and collected a series of nine further cases from the literature of the subject. Most of these began, according to them, in the manner of an infection, four cases having throat inflammation, three necrosis of the jaw or palate. The lymphatic glands may be small or moderately enlarged, the spleen palpable, not much enlarged, hæmorrhages are constant, and there is an irregular low fever. The blood is as follows:—

(1) Anæmia progressive and severe.

(2) A large increase of white blood corpuscles.

(3) A large proportion, 25-96 per cent., of the white cells made up of myelocytes, large mononuclear cells of the same size, and faintly granular large mononuclear clear cells (transitional).

(4) Eosinophiles, mast cells, and nucleated red cells may be absent or present in varying quantities.

Billings and Cap's case showed 54 per cent. of myelocytes.

II. *Chronic spleno-medullary leukæmia.*—This is the common form of the disease, cases cropping up pretty frequently in the wards of any of the larger hospitals. Patients usually come complaining of weakness and loss of health, or on account of their abdomen swelling, or on account of shortness of breath, and so on. The symptoms are those of anæmia, with, in addition, a great enlargement of the spleen. Hæmorrhages are common, there is generally a low type of irregular fever, later there may be ascites, but note specially there is no enlargement of glands. Clinically this condition is simulated by another disease, splenic anæmia (*vide* later), but the blood examination at once differentiates them. The changes found there summarised are as follows:—

(1) Anæmia of a fairly severe grade, two to three million reds.

(2) An enormous number of white cells, 300,000 to 500,000 on an average.

(3) A large proportion of the white cells present made up of myelocytes 30 to 40 per cent., polymorphonuclears fairly numerous, lymphocytes and eosinophiles relatively diminished. Many cells intermediate between the different standard types and which are not easy to classify.

(4) Many nucleated red cells (chiefly normoblasts).

The following is an actual count:—

| | | |
|---|---|---|
| R. | 2,890,000 | 15 nucleated reds seen in counting 1,000 whites. Reds slight poikilocytosis, but not very marked. |
| W. | 352,000 | |
| Hb. | 50 % | |

Differential:

| | Per cent. | |
|---|---|---|
| Polymorphonuclear ... | 38 | 1,000 counted |
| Large mononuclear ... | 0·5 | |
| Lymphocytes ... ... | 2 | |
| Eosinophile ... ... | 0·5 | |
| Transitional ... ... | 1 | |
| Mast cells ... ... | 2 | |
| Myelocytes ... ... | 56 | |
| | 100 | |

The diagnosis, once the blood examination is made, is very simple. The prognosis is not good, though since the introduction of $x$-rays in the treatment results are more hopeful. This causes the blood to return to a more normal condition, but if the treatment is stopped the number of whites begins to rise again, and it is doubtful if myelocytes ever disappear.

III. *Acute lymphatic leukæmia.*—This condition is not very common. It is usual to consider cases in which large lymphocytes predominate as acute, those in which small predominate as chronic, but this distinction does not always hold, a third group sometimes being seen in which both types are found. The symptoms of an acute case are something as follows: There is anæmia, generally an irregular fever, vomiting and diarrhœa, and numerous hæmorrhages, such as epistaxis, purpura, hæmatemesis, etc. The lymphatic glands all over the body are enlarged, and the spleen also. In the blood over 90 per cent. of the leucocytes present will be large lymphocytes, and there will be severe anæmia.

IV. *Chronic lymphatic leukæmia.*—This form, though generally spoken of as chronic and associated with small lymphocytes, may nevertheless run a very acute course. The following is the count of a case who died very quickly after the first signs of the disease were noted. The count brings out all the points seen in the blood examination.

| | | |
|---|---|---|
| R. | 1,870,000 | Well-marked poikilocytosis, nucleated reds; two normoblasts seen. |
| W. | 135,800 | |
| Hb. | 40 % | |

Differential:

| | Per cent | |
|---|---|---|
| Polymorphonuclear ... | 1 | 1,000 counted. |
| Large mononuclear ... | 0·3 | |
| Lymphocytes ... ... | 98·7 | |
| Eosinophile ... ... | 0 | |
| Transitional ... ... | 0 | |
| Mast cells ... ... | 0 | |
| | 100 | |

These changes summarised are as follows:—

(1) Severe anæmia, nucleated reds not common.

(2) Whites, over 100,000.

(3) Lymphocytes, 98 per cent. of total leucocytes.

(4) Other usual leucocytes practically absent.

Clinically, there was also a marked enlargement of all the lymphatic glands all over the body, and the spleen was considerably enlarged, being well over 1 lb. in weight.

Two other diseases may also be mentioned here—namely, splenic anæmia and Hodgkin's disease. In the latter the blood is normal, in the former we find a severe anæmia (two million or under) of a secondary type, but with the leucocytes normal or diminished.

An actual count gave:—

| | | |
|---|---|---|
| R. | 2,300,000 | A few nucleated reds seen; poikilocytosis slight, if any. No megalocytes. |
| W. | 4,000 | |
| Hb. | 45 % | |

Differential count, nothing abnormal.

In addition to the anæmia, the spleen in this disease is very much enlarged (often as big as a leukæmic spleen), but the lymphatic glands are normal. The only way to tell it from leukæmia is by examining the blood. In the tropical disease, kala-azar, a very similar blood picture is found. An anæmia of a secondary type, a very low leucocyte count, and not much poikilocytosis. Clinically, there is also a big spleen, cachexia, and very often hæmorrhages from different parts of the body, while the lymphatic glands are also normal. The ultimate diagnosis is reached by puncturing the liver and demonstrating the Leishman-Donovan bodies.

